# MicroRNA dysregulation in uveal melanoma: a new player enters the game

**DOI:** 10.18632/oncotarget.2923

**Published:** 2015-02-19

**Authors:** Zheng Li, Xin Yu, Jianxiong Shen, Yang Jiang

**Affiliations:** ^1^ Department of Orthopaedic Surgery, Peking Union Medical College Hospital, Chinese Academy of Medical Sciences and Peking Union Medical College, Beijing 100730, China; ^2^ Department of Ophthalmology, Peking Union Medical College Hospital, Chinese Academy of Medical Sciences and Peking Union Medical College, Beijing 100730, China

**Keywords:** Uveal melanoma, microRNAs, diagnosis and therapy, oncogene, tumor suppressor

## Abstract

Uveal melanoma is the second most common form of melanoma and a predominant intraocular malignant tumor in adults. The development of uveal melanoma is a multistep process involving genetic and epigenetic alteration of proto-oncogenes and tumor-suppressor genes. Recent discoveries have shed a new light on the involvement of a class of noncoding RNA known as microRNAs (miRNAs) in uveal melanoma. A lot of miRNAs show differential expressions in uveal melanoma tissues and cell lines. Genes coding for these miRNAs have been characterized as novel oncogene and tumor-suppressor genes based on findings that these miRNAs control malignant phenotypes of uveal melanoma cells. Several studies have confirmed that dysregulation of miRNAs promotes cell-cycle progression, confers resistance to apoptosis, and enhances invasiveness and metastasis. Moreover, several miRNAs have also been shown to correlate with uveal melanoma initiation and progression, and thus may be used as biomarkers for early diagnosis and prognosis. Elucidating the biological aspects of miRNA dysregulation may help us better understand the pathogenesis of uveal melanoma and promote the development of miRNA directed-therapeutics against this disease.

## INTRODUCTION

Uveal melanoma is the second most common form of melanoma and the most common primary intraocular malignant tumor in adults, with an incidence of about 1200–1500 new cases per year in the United States, accounting for 5%–6% of all cases of primary systemic melanoma [1–4]. Uveal melanoma is one of the most highly aggressive cancer and leada to metastatic death in up to half of patients despite successful local therapy [5, 6]. Early metastasis accounts for the high death rate of uveal melanoma [7]. Unfortunately, many patients have subclinical metastasis at the time of diagnosis [8]. Despite the advances in surgery, chemotherapy and radiotherapy, the 5-year relative survival rate has not improved from 1973 to 2008 [9–11]. Metastasis of uveal melanoma is a complex and multistep process, involving increased proliferative, migratory and invasive potential of tumor cells [12]. Currently, the molecular mechanisms of its aggressiveness are still not elucidated [13, 14]. Therefore, identifying the crucial signals that promote invasive and metastatic potential of uveal melanoma will contribute to identify biomarkers for early diagnosis and targets for treatment.

MicroRNAs (miRNAs) are a recently discovered class of short (17–22 nucleotides in length), endogenous, and noncoding RNAs that regulate gene expression and thereby play significant roles in human development and various pathological conditions [15–18]. By base-pairing with the complementary sites in the 3′untranslated region (3′UTR) of the mRNA, miRNAs regulate target genes by increasing mRNA decay or repressing translation [19–21]. Growing evidences indicate that miRNAs control many cellular processes, including cell development, differentiation, proliferation and apoptosis [22–27]. Abnormal miRNA expression has been found in many human tumors, including colorectal, bladder cancer, hepatocellular carcinoma, gastric and breast cancers [28–32]. This review will focus on recent discoveries related to the miRNAs involved in the development of uveal melanoma and discuss the potential use of miRNAs as diagnostic and prognostic biomarkers and treatment strategies for uveal melanoma.

### Function and biogenesis of miRNA

MiRNAs were initially discovered in *Caenorhabditis elegans* in 1993, but were not found their existence in mammals until 2000 [33, 34]. To date, more than 1500 miRNAs have been identified in humans [35]. A single miRNA can downregulate multiple targets, which often belong to the same metabolic or signaling pathway [36, 37]. It is estimated that miRNAs could regulate the expression of at least 20%–30% of all human genes [38].

The primary miRNA transcript is also called pri-miRNA, which is transcribed by RNA polymerase II or III [39]. The pri-miRNA is then cleaved by the Drosha-DGCR8 microprocessor complex to produce the precursor hairpin molecule (pre-miRNA) which is then transferred from the nucleus to the cytoplasm by exportin25/Ran-GTP [40]. With the assistance of a complex that contains the RNase Dicer and the double-stranded RNA-binding protein, TRBP, the 70-nucleotide pre-miRNA is refined into mature miRNA [41]. The functional strand of the mature miRNA is located on the RNA-induced silencing complex (RISC), which contains the proteins, argonaute (Ago) and Tnrc6, while the other strand is usually degraded [42]. The mature miRNA guides the RISC to the imperfect complementary sequences in target mRNAs to promote transcript decay, repress the cognate mRNA translation, or both [43].

### Alteration of miRNAs in uveal melanoma

A number of expression profiling studies have demonstrated miRNAs were dysregulated in uveal melanoma. *Worley* et al. determined and compared the expression of 470 microRNAs in 24 primary uveal melanomas [44]. Tumors readily clustered based on miRNA expression into two groups that corresponded to the gene expression-based subtypes: class 1 (low metastatic risk) and class 2 (high metastatic risk). The most significant discriminators were let-7b and miR-199a, and the expression of these miRNAs was further validated by quantitative PCR. A classifier that included the top six miRNA discriminators accurately distinguished class 1 from class 2 tumors with 100% sensitivity and specificity. *Yan* et al. demonstrated that miR-34a was significantly down-expressed in uveal melanomas cell lines and tissues compared with the melanocytes using northern blot analysis [45]. A study found that 19 miRNAs expressed in non-metastasizing melanoma were absent in metastasizing melanoma; 11 miRNAs were found to be expressed in metastasizing melanoma and absent in non-metastasizing melanoma, as listed in Table 1 [46]. In addition, it was found that miR-137 expression was lower in uveal melanoma cell lines than in uveal melanocytes using Real-time RT-PCR analysis [47]. Using miRNA microarray analysis, Yang et al. demonstrated that miRNA-20a, miRNA-106a, miRNA-17, miRNA-21, and miRNA-34a were up-regulated, while miRNA-145 and miRNA-204 expression were down-regulated in four uveal melanoma tissues [48]. *Dong* et al. demonstrated that miR-34b/c expression was dramatically decreased in uveal melanoma cells and clinical samples [49]. MiR-9 was significantly reduced in highly invasive uveal melanoma cell lines [50]. Chen et al. determined that miR-124a expression was down-regulated in both uveal melanoma cells and clinical specimens [51]. Another study also found that 59 most varying miRNAs was detected in 26 uveal melanomas [52]. However, no significant association of miRNA clusters with TNM stages was observed. *Venza* et al. determined the miRNA signatures in the cutaneous melanoma cell line G361 and the uveal melanoma cell line OCM-1 [53]. They identified 96 miRNAs that were modified in both cell models. Among these commonly modified miRNAs, 65 were down-regulated, 28 up-regulated, and 3 exhibited a different expression trend. Li et al. also found that 47 miRNAs were up-regulated in uveal melanoma and 61 were down-regulated in their study on miRNAs expression profile in uveal melanoma sample [54]. Achberger et al. found that plasma levels of miR-20a, 125b, 146a, 155, 181a, and 223 were higher in the study patients at diagnosis as uveal melanoma compared to controls. Plasma levels of miR-20a, 125b, 146a, 155, and 223 increased, and miR-181a decreased when metastasis manifested [55].

**Table 1:** MiRNA expression profiles in uveal melanoma (UM)

Num	Method	Sample	Upregulated	Downregulated	Reference
1	Microarray	primary UM	let-7b, miR-199a, miR-199a*, miR-143, miR-193b, and miR-652 (high metastatic risk)		44
2	Microarray	primary UM	miR-549, miR-497, miR-885-5p, miR-585, miR-640, miR-512-5p, miR-556-5p, miR-135b, miR-325, miR-99a, miR-33a, (high metastatic risk)	miR-495, miR-18a, miR-586, miR-493, miR-377, miR-376c, miR-269-3p, miR-34c-5p, miR-26a-2, miR-218, miR-19b-1, miR-181a, miR-154, miR-133a, miR-129, miR-10a, miR-1, Let-7e	46
3	Microarray RT-PCR	primary UM	miRNA-20a, miRNA-106a, miRNA-17, miRNA-21, miRNA-34a	miRNA-145, miRNA-204	48
4	Microarray	UM cell lines epidermal melanocytes	28 miRNAs	65 miRNAs	53
5	Microarray	Primary UM	47 miRNAs	61 miRNAs	54

However, only a small number of miRNA expressions were shared among different studies and several miRNAs even exhibit discordant expression patterns among these studies. These discrepancies are probably due to quality of clinical samples, the indistinctive change and specificity of profiling platforms, different protocols for sample collection and processing, preceding cytotoxic treatments, tumor heterogeneity and underestimated hypoxia and infection. Thus, it is important to reevaluate current strategies in miRNA profiling and be cautious about the interpretation of those existing signatures.

### Biological functions of deregulated miRNAs in uveal melanoma

Since increasing deregulated miRNAs were demonstrated, further functional characterization of these miRNAs, especially their interaction with oncogenes, tumor suppressor genes and other cancer-related genes, is important for us to understand the molecular tumorigenesis of uveal melanoma. For example, functional analysis of miR-34a in uveal melanoma cell lines indicated that miR-34a was inhibited the proliferative ability and migration of uveal melanoma cells. Moreover, bioinformatic prediction suggested that the oncogene, c-Met, was a target gene of miR-34a in uveal melanoma cells. Furthermore, miR-34a down-regulated phosphorylated Akt and cell cycle-related proteins [45]. Another study showed that miR-34b/c expression was dramatically decreased in uveal melanoma cells and clinical samples, which can be upregulated by doxorubicin and epigenetic drugs. The transfection of miR-34b/c into uveal melanoma cells also leads to a significant reduction in cell growth and migration. miR-34b/c caused cell cycle G(1) arrest rather than the induction of apoptosis. Met proto-oncogene (c-Met) was identified as a target of miR-34b/c in uveal melanoma cells. Furthermore, miR-34b/c was confirmed to downregulate the expression of c-Met, p-Akt, and cell cycle-related proteins by western blotting [49]. Genistein, an isoflavone isolated from soybean, has been found to be a potent antitumor agent [56]. Genistein markedly inhibited miR-27a expression in a concentration-dependent manner and BTB domain containing 10 (ZBTB10) was proved to the target of miR-27a. It is biologically plausible that the decrease of miR-27a expression resulting in post-transcriptional gene regulation in uveal melanoma cells might partly account for the inhibitive effect of genistein on human uveal melanoma [56]. *Chen* et al. demonstrated that miR-137 expression was lower in uveal melanoma cell lines than in uveal melanocytes [47]. Functional analysis of miR-137 in uveal melanoma cell lines indicated that over-expression of miR-137 induced G1 cell cycle arrest, leading to a significant decrease in cell growth in uveal melanoma cells. Ectopic transfection of miR-137 into uveal melanoma cells downregulated MITF, a transcription factor with oncogenic activity. Moreover, overexpression of miR-137 downregulated the oncogenic tyrosine kinase protein receptor c-Met and cell cycle-related proteins, including CDK6. Liu et al. reported that the expression of miR-9 was significantly reduced in highly invasive uveal melanoma cell lines, and miR-9 overexpression suppressed migration and invasion of highly invasive cells [50]. Furthermore, miR-9 negatively modulated NF-κB1 expression by directly targeting its 3′-UTRs. Additionally, downstream targets of NF-κB1, such as MMP-2, MMP-9 and VEGFA, were regulated by miR-9 in the same pattern as NF-κB1. Therefore, miR-9 suppressed uveal melanoma cell migration and invasion partly through downregulation of the NF-κB1 signaling pathway. Yan et al. demonstrated that miR-182 expression was dependent on p53 induction in uveal melanoma cells. [57] Transient transfection of miR-182 into cultured uveal melanoma cells led to a significant decrease in cell growth, migration, and invasion. Cells transfected with miR-182 demonstrated cell cycle G1 arrest and increased apoptotic activity. MiR-182 was proved to exert its role on mRNA expression by targeting the 3′ untranslated region of MITF, BCL2 and cyclin D2. The expression of oncogene c-Met and its downstream Akt and ERK1/2 pathways were also downregulated by miR-182. Concordant with the findings that miR-182 was decreased in uveal melanoma tissue samples, overexpression of miR-182 suppressed the growth of uveal melanoma cells *in vivo*. Previous study has demonstrated that miR-124a expression was downregulated in both uveal melanoma cells and clinical specimens [51]. Transient transfection of miR-124a into uveal melanoma cells inhibited cell growth, migration, and invasion. Moreover, Overexpression of miR-124a suppressed *in vivo* growth of tumor. Potential targets of miR-124a included CDK4, CDK6, cyclin D2, and EZH2. Recently study shwed that miR-145 expression was significantly lower in uveal melanoma sample and cell lines were compared with normal uveal sample. Overexpression of miR-145 suppressed cell proliferation by blocking the G1 phase entering S phase in uveal melanoma cells, and promoted uveal melanoma cell apoptosis. IRS-1 was identified as a potential target of miR-145 by dual luciferase reporter assay (Table 2) [54].

**Table 2:** Functional characterization of the deregulated miRNAs in uveal melanoma (UM)

Name	Up or down regulation	Target gene	Role	Reference
miR-34a	Down	c-Met	Tumor suppressor	45
miR-137	Down	MITF, c-Met, CDK6	Tumor suppressor	47
miR-34b/c	Down	c-Met	Tumor suppressor	49
miR-9	Down	NF-κB1	Tumor suppressor	50
miR-124a	Down	CDK4, CDK6, cyclinD2, EZH2	Tumor suppressor	51
miR-145	Down	IRS-1	Tumor suppressor	54
miR-27a	Up	ZBTB10	oncogene	56
miR-182	Down	MITF, BCL2, cyclin D2, c-Met	Tumor suppressor	57

### Prognostic use of miRNAs and other clinical implications

Larsen et al. detected miRNAs expression in 36 patients with uveal melanoma. The miRNAs hierarchical clustering divided the uveal melanoma into three groups based on microRNA expression. The clusters showed no association with clinical or histopathological features, TNM staging, metastasis or survival in uveal melanoma patients [52]. Differential expression analysis did not reveal microRNAs related to metastasis or survival. To date, despite overwhelming reports of dysregulated miRNAs in uveal melanoma tissues, no circulating miRNA has been identified for non-invasive diagnosis of uveal melanoma.

### Mechanisms of miRNA deregulation in uveal melanoma

The mechanisms of miRNA deregulation in cancer are complex. The miRNA genes are regulated in similar way with other coding genes. Recent works have provided new insights to explain miRNA deregulation in uveal melanoma, including epigenetic alteration and deregulated transcription. Previous study showed that genistein markedly inhibited miR-27a expression and enhanced its target gene ZBTB10 expression in uveal melanoma [56]. miR-137 may be epigenetically silenced during uveal melanoma tumorigenesis. Chen et al. reported that a DNA hypomethylating agent, 5-aza-2′-deoxycytidine, could increase the expression levels of miR-137 [47]. In addition, miR-124a expression was found to be regulated via epigenetic mechanisms, with its expression restored when cells were treated with a DNA hypomethylating agent, 5-aza-2′-deoxycytidine, and a histone deacetylase inhibitor, trichostatin A [51].

### Conclusions and future perspectives

Uveal melanoma is one of the most highly aggressive cancer that leads to metastatic death in up to half of patients despite successful local therapy [58]. Early metastasis accounts for the high death rate of uveal melanoma [59]. However, early diagnosis of uveal melanoma is a major challenge because of the lack of knowledge about the molecular mechanisms of uveal melanoma [60]. Therefore, a more comprehensive understanding of the pathogenic mechanism of uveal melanoma is useful for formulating innovative therapeutic strategies [61]. Dysregulation of miRNA occurs in uveal melanoma as well as other malignant diseases [39]. The mechanisms of miRNA in tumor development and progression are complex and numerous [62]. However, most of them converge on common signaling mechanisms that govern cell proliferation, apoptosis and invasiveness [63]. Moreover, the significance of specific miRNAs in uveal melanoma development should be interpreted in appropriate biological contexts as miRNA interacts widely with other signaling cascades and may behave differently in different histological subtypes of uveal melanoma. Population-based differences in miRNA dysregulation, and thus the diagnostic or prognostic use of miRNAs in different ethnic groups are also key considerations [64]. Recent advances in the development of *in vivo* RNA delivery system may open a new window for use of miRNA as new cancer therapeutics [65]. In addition, miRNAs may be targeted by a novel class of chemically engineered oligonucleotides known as antagomirs that silence endogenous miRNAs [66]. It is anticipated that, with a more comprehensive understanding of miRNA dysregulation and the associated abnormalities in cellular signaling in uveal melanoma, novel therapeutics will emerge.

## References

[1] Rashid AB, Grossniklaus HE (2014). Clinical, pathologic, and imaging features and biological markers of uveal melanoma. Methods Mol Biol.

[2] Yousef YA, Alkilany M (2014). Characterization, treatment, and outcome of uveal melanoma in the first two years of life. Hematol Oncol Stem Cell Ther.

[3] Yonekawa Y, Kim IK (2012). Epidemiology and management of uveal melanoma. Hematol Oncol Clin North Am.

[4] Blanco PL, Lim LA, Miyamoto C, Burnier MN (2012). Uveal melanoma dormancy: an acceptable clinical endpoint?. Melanoma Res.

[5] Schoenfield L (2014). Uveal melanoma: A pathologist's perspective and review of translational developments. Advances in anatomic pathology.

[6] Gill HS, Char DH (2012). Uveal melanoma prognostication: from lesion size and cell type to molecular class. Can J Ophthalmol.

[7] Luke JJ, Callahan MK, Postow MA, Romano E, Ramaiya N, Bluth M, Giobbie-Hurder A, Lawrence DP, Ibrahim N, Ott PA, Flaherty KT, Sullivan RJ, Harding JJ, D'Angelo S, Dickson M, Schwartz GK (2013). Clinical activity of ipilimumab for metastatic uveal melanoma: a retrospective review of the Dana-Farber Cancer Institute, Massachusetts General Hospital, Memorial Sloan-Kettering Cancer Center, and University Hospital of Lausanne experience. Cancer.

[8] Pereira PR, Odashiro AN, Lim LA, Miyamoto C, Blanco PL, Odashiro M, Maloney S, De Souza DF, Burnier MN (2013). Current and emerging treatment options for uveal melanoma. Clin Ophthalmol.

[9] Eschelman DJ, Gonsalves CF, Sato T (2013). Transhepatic therapies for metastatic uveal melanoma. Semin Intervent Radiol.

[10] Buder K, Gesierich A, Gelbrich G, Goebeler M (2013). Systemic treatment of metastatic uveal melanoma: review of literature and future perspectives. Cancer medicine.

[11] Velho TR, Kapiteijn E, Jager MJ (2012). New therapeutic agents in uveal melanoma. Anticancer research.

[12] Patel SP (2013). Latest developments in the biology and management of uveal melanoma. Current oncology reports.

[13] Coupland SE, Lake SL, Zeschnigk M, Damato BE (2013). Molecular pathology of uveal melanoma. Eye (Lond).

[14] Griewank KG, Murali R (2013). Pathology and genetics of uveal melanoma. Pathology.

[15] Ohdaira H, Sekiguchi M, Miyata K, Yoshida K (2012). MicroRNA-494 suppresses cell proliferation and induces senescence in A549 lung cancer cells. Cell proliferation.

[16] Fu LL, Zhao X, Xu HL, Wen X, Wang SY, Liu B, Bao JK, Wei YQ (2012). Identification of microRNA-regulated autophagic pathways in plant lectin-induced cancer cell death. Cell proliferation.

[17] Li J, You T, Jing J (2014). MiR-125b inhibits cell biological progression of Ewing's sarcoma by suppressing the PI3K/Akt signalling pathway. Cell proliferation.

[18] Li M, Yu M, Liu C, Zhu H, He X, Peng S, Hua J (2013). miR-34c works downstream of p53 leading to dairy goat male germline stem-cell (mGSCs) apoptosis. Cell proliferation.

[19] Zhou J, Song S, Cen J, Zhu D, Li D, Zhang Z (2012). MicroRNA-375 is downregulated in pancreatic cancer and inhibits cell proliferation *in vitro*. Oncology research.

[20] Wang Z, Yin B, Wang B, Ma Z, Liu W, Lv G (2014). MicroRNA-0 promotes proliferation and invasion of peripheral nerve sheath tumor cells targeting EFNA3. Oncology research.

[21] Fei B, Wu H (2012). MiR-378 inhibits progression of human gastric cancer MGC-803 cells by targeting MAPK1 *in vitro*. Oncology research.

[22] Huang J, Zhang SY, Gao YM, Liu YF, Liu YB, Zhao ZG, Yang K (2014). MicroRNAs as oncogenes or tumour suppressors in oesophageal cancer: potential biomarkers and therapeutic targets. Cell proliferation.

[23] Wang SC, Lin XL, Li J, Zhang TT, Wang HY, Shi JW, Yang S, Zhao WT, Xie RY, Wei F, Qin YJ, Chen L, Yang J, Yao KT, Xiao D (2014). MicroRNA-122 triggers mesenchymal-epithelial transition and suppresses hepatocellular carcinoma cell motility and invasion by targeting RhoA. PloS one.

[24] Xu Y, Jin J, Liu Y, Huang Z, Deng Y, You T, Zhou T, Si J, Zhuo W (2014). Snail-regulated MiR-375 inhibits migration and invasion of gastric cancer cells by targeting JAK2. PloS one.

[25] Shen J, Niu W, Zhou M, Zhang H, Ma J, Wang L (2014). MicroRNA-410 suppresses migration and invasion by targeting MDM2 in gastric cancer. PloS one.

[26] Ninio-Many Lihi, Grossman Hadas, Levi Mattan, Zilber Sofia, Tsarfaty Ilan, Shomron Noam, Tuvar Anna, Chuderl Dana, Stemmer Salomon M, Ben-Aharon Irit, Shalgi Ruth (2014). MicroRNA miR-125a-3p modulates molecular pathway of motility and migration in prostate cancer cells. Oncoscience.

[27] Sidiropoulos Konstantinos G., White Nicole M.A., Bui Anna, Ding Qiang, Boulos Peter, Pampalakis Georgios, Khella Heba, Samuel Joseph N, Sotiropoulou Georgia, Yousef George M (2014). Kallikrein-related peptidase 5 induces miRNA-mediated anti-oncogenic pathways in breast cancer. Oncoscience.

[28] Xiong X, Ren HZ, Li MH, Mei JH, Wen JF, Zheng CL (2011). Down-regulated miRNA-214 induces a cell cycle G1 arrest in gastric cancer cells by up-regulating the PTEN protein. Pathol Oncol Res.

[29] Wang Z, Wang N, Liu P, Chen Q, Situ H, Xie T, Zhang J, Peng C, Lin Y, Chen J (2014). MicroRNA-25 regulates chemoresistance-associated autophagy in breast cancer cells, a process modulated by the natural autophagy inducer isoliquiritigenin. Oncotarget.

[30] Yang WB, Chen PH, Hsu Ts, Fu TF, Su WC, Liaw H, Chang WC, Hung JJ (2014). Sp1-mediated microRNA-182 expression regulates lung cancer progression. Oncotarget.

[31] Zhang C, Liu J, Wang X, Wu R, Lin M, Laddha SV, Yang Q, Chan CS, Feng Z (2014). MicroRNA-339-p inhibits colorectal tumorigenesis through regulation of the MDM2/p3 signaling. Oncotarget.

[32] Hung TM, Ho CM, Liu YC, Lee JL, Liao YR, Wu YM, Ho MC, Chen CH, Lai HS, Lee PH (2014). Up-regulation of microRNA-10b plays a role for decreased IGF-1 that induces insulin resistance in human hepatocellular carcinoma. PloS one.

[33] Lee RC, Feinbaum RL, Ambros V (1993). The C. elegans heterochronic gene lin-4 encodes small RNAs with antisense complementarity to lin-14. Cell.

[34] Kong YW, Ferland-McCollough D, Jackson TJ, Bushell M (2012). microRNAs in cancer management. The Lancet Oncology.

[35] Dassow H, Aigner A (2013). MicroRNAs (miRNAs) in colorectal cancer: from aberrant expression towards therapy. Current pharmaceutical design.

[36] Yu X, Li Z, Shen J, Wu WK, Liang J, Weng X, Qiu G (2013). MicroRNA-10b Promotes Nucleus Pulposus Cell Proliferation through RhoC-Akt Pathway by Targeting HOXD10 in Intervetebral Disc Degeneration. PloS one.

[37] Li Z, Lei H, Luo M, Wang Y, Dong L, Ma Y, Liu C, Song W, Wang F, Zhang J, Shen J, Yu J (2015). DNA methylation downregulated mir-10b acts as a tumor suppressor in gastric cancer. Gastric cancer : official journal of the International Gastric Cancer Association and the Japanese Gastric Cancer Association.

[38] Wu WK, Lee CW, Cho CH, Fan D, Wu K, Yu J, Sung JJ (2010). MicroRNA dysregulation in gastric cancer: a new player enters the game. Oncogene.

[39] Stark MS, Tyagi S, Nancarrow DJ, Boyle GM, Cook AL, Whiteman DC, Parsons PG, Schmidt C, Sturm RA, Hayward NK (2010). Characterization of the Melanoma miRNAome by Deep Sequencing. PloS one.

[40] Wu WK, Law PT, Lee CW, Cho CH, Fan D, Wu K, Yu J, Sung JJ (2011). MicroRNA in colorectal cancer: from benchtop to bedside. Carcinogenesis.

[41] Zhao J, Kelnar K, Bader AG (2014). In-depth analysis shows synergy between erlotinib and miR-34a. PloS one.

[42] Morishita A, Masaki T (2014). miRNA in hepatocellular carcinoma. Hepatology research : the official journal of the Japan Society of Hepatology.

[43] Liang W, Gao B, Fu P, Xu S, Qian Y, Fu Q (2013). The miRNAs in the pathgenesis of osteosarcoma. Front Biosci (Landmark Ed).

[44] Worley LA, Long MD, Onken MD, Harbour JW (2008). Micro-RNAs associated with metastasis in uveal melanoma identified by multiplexed microarray profiling. Melanoma Res.

[45] Yan D, Zhou X, Chen X, Hu DN, Dong XD, Wang J, Lu F, Tu L, Qu J (2009). MicroRNA-34a inhibits uveal melanoma cell proliferation and migration through downregulation of c-Met. Investigative ophthalmology & visual science.

[46] Radhakrishnan A, Badhrinarayanan N, Biswas J, Krishnakumar S (2009). Analysis of chromosomal aberration and association of microRNAs in uveal melanoma. Molecular vision.

[47] Chen X, Wang J, Shen H, Lu J, Li C, Hu DN, Dong XD, Yan D, Tu L (2011). Epigenetics, microRNAs, and carcinogenesis: functional role of microRNA-137 in uveal melanoma. Investigative ophthalmology & visual science.

[48] Yang C, Wei W (2011). The miRNA expression profile of the uveal melanoma. Sci China Life Sci.

[49] Dong F, Lou D (2012). MicroRNA-34b/c suppresses uveal melanoma cell proliferation and migration through multiple targets. Molecular vision.

[50] Liu N, Sun Q, Chen J, Li J, Zeng Y, Zhai S, Li P, Wang B, Wang X (2012). MicroRNA-9 suppresses uveal melanoma cell migration and invasion through the NF-kappaB1 pathway. Oncology reports.

[51] Chen X, He D, Dong XD, Dong F, Wang J, Wang L, Tang J, Hu DN, Yan D, Tu L (2013). MicroRNA-124a is epigenetically regulated and acts as a tumor suppressor by controlling multiple targets in uveal melanoma. Investigative ophthalmology & visual science.

[52] Larsen AC, Holst L, Kaczkowski B, Andersen MT, Manfe V, Siersma VD, Kolko M, Kiilgaard JF, Winther O, Prause JU, Gniadecki R, Heegaard S (2014). MicroRNA expression analysis and Multiplex ligation-dependent probe amplification in metastatic and non-metastatic uveal melanoma. Acta Ophthalmol.

[53] Venza M, Dell'Aversana C, Visalli M, Altucci L, Teti D, Venza I (2014). Identification of microRNA expression patterns in cutaneous and uveal melanoma cell lines. Tumori.

[54] Li Y, Huang Q, Shi X, Jin X, Shen L, Xu X, Wei W (2014). MicroRNA 145 may play an important role in uveal melanoma cell growth by potentially targeting insulin receptor substrate-1. Chinese medical journal.

[55] Achberger S, Aldrich W, Tubbs R, Crabb JW, Singh AD, Triozzi PL (2014). Circulating immune cell and microRNA in patients with uveal melanoma developing metastatic disease. Mol Immunol.

[56] Sun Q, Cong R, Yan H, Gu H, Zeng Y, Liu N, Chen J, Wang B (2009). Genistein inhibits growth of human uveal melanoma cells and affects microRNA-27a and target gene expression. Oncology reports.

[57] Yan D, Dong XD, Chen X, Yao S, Wang L, Wang J, Wang C, Hu DN, Qu J, Tu L (2012). Role of microRNA-182 in posterior uveal melanoma: regulation of tumor development through MITF, BCL2 and cyclin D2. PloS one.

[58] Shildkrot Y, Thomas F, Al-Hariri A, Fry CL, Haik BG, Wilson MW (2011). Socioeconomic factors and diagnosis of uveal melanoma in the mid-southern United States. Curr Eye Res.

[59] Singh AD, Turell ME, Topham AK (2011). Uveal melanoma: trends in incidence, treatment, and survival. Ophthalmology.

[60] Damato B (2010). Does ocular treatment of uveal melanoma influence survival?. British journal of cancer.

[61] Augsburger JJ, Correa ZM, Shaikh AH (2009). Effectiveness of treatments for metastatic uveal melanoma. American journal of ophthalmology.

[62] Liu HS, Xiao HS (2014). MicroRNAs as potential biomarkers for gastric cancer. World journal of gastroenterology : WJG.

[63] Banaudha KK, Verma M (2012). The role of microRNAs in the management of liver cancer. Methods Mol Biol.

[64] Wang J, Wang Q, Liu H, Hu B, Zhou W, Cheng Y (2010). MicroRNA expression and its implication for the diagnosis and therapeutic strategies of gastric cancer. Cancer letters.

[65] Li X, Yang W, Lou L, Chen Y, Wu S, Ding G (2014). microRNA: a promising diagnostic biomarker and therapeutic target for hepatocellular carcinoma. Digestive diseases and sciences.

[66] Tsujiura M, Ichikawa D, Komatsu S, Shiozaki A, Takeshita H, Kosuga T, Konishi H, Morimura R, Deguchi K, Fujiwara H, Okamoto K, Otsuji E (2010). Circulating microRNAs in plasma of patients with gastric cancers. British journal of cancer.

